# Supplementary material for: Controlling Mixed Mo/MoS2 Domains on Si by Molecular Beam Epitaxy for the Hydrogen Evolution Reaction
Source: ACS Nano. 2026 Jan 27;20(5):4479–93. doi: 10.1021/acsnano.5c19478 (PMC12895511; doi:10.1021/acsnano.5c19478)
Supplement: Supplementary file 1 [file nn5c19478_si_001.pdf]

Supplementary Information

Controlling Mixed Mo/MoS_2 Domains on Si by Molecular Beam Epitaxy for the Hydrogen Evolution Reaction

Eunseo Jeon[1,*], Vincent Masika Peheliwa[2,3,*], Marie Hrůzová Kratochvílová[2], Tim Verhagen[2,†], Yong-Kul Lee[1,†]

[1]Laboratory of Advanced Catalysis for Energy and Environment, Department of Chemical Engineering, Dankook University, Yongin 16890, South Korea

[2]Institute of Physics of the Czech Academy of Sciences, Prague 182 00, Czech Republic

[3]Faculty of Mathematics and Physics, Charles University, Prague 121 16, Czech Republic

* Equally contributed to this work

† Email: verhagen@fzu.cz; yolee@dankook.ac.kr

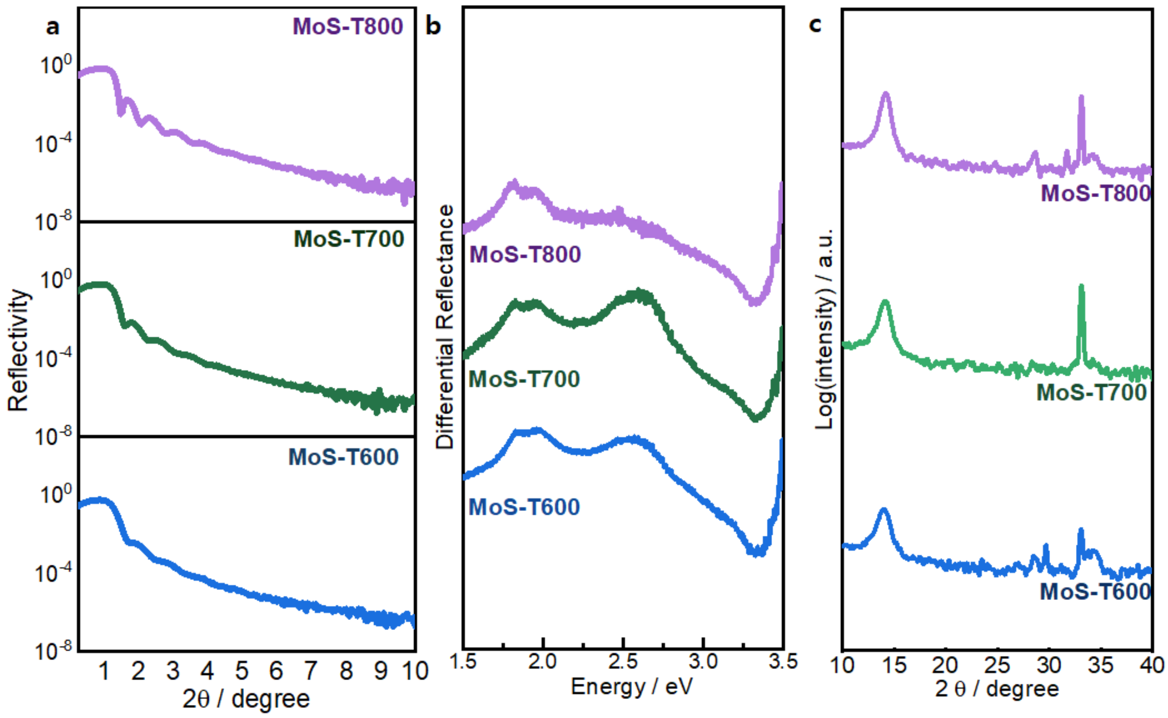

Figure S1. (a, b, c) Structural evolution of MBE-grown MoS_2: XRR patterns, differential reflectance spectra, and log-scaled XRD pattern of MoS_2 thin films grown with different annealing temperature.

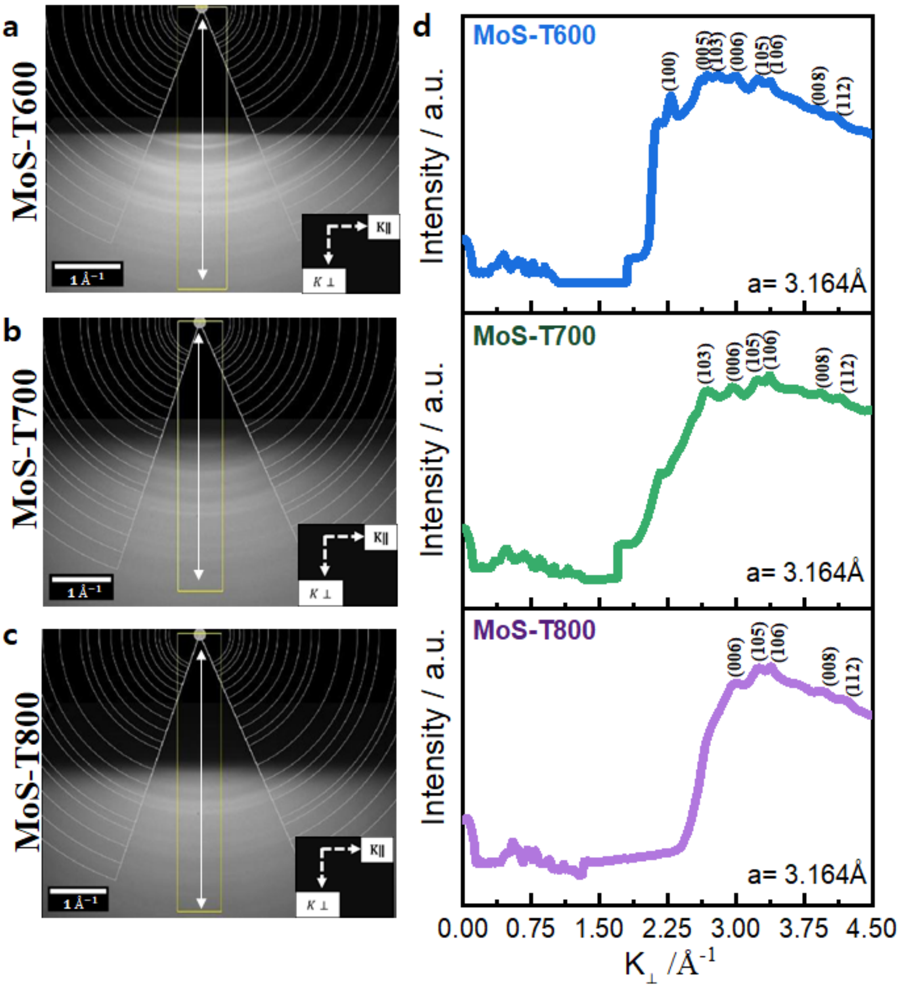

Figure S2. (a-d) Structural and surface characterization of MoS_2 thin films. RHEED patterns for MoS-T600 (a), T700 (b), and T800 (c) after annealing at 20 °C. (d) Intensity profiles as a function of momentum transfer perpendicular to the substrate $K_\perp$ along the white line shown in (a-c). The visible peaks are labeled according to the 2H-MoS_2 structure and the lattice parameter *a* is determined.

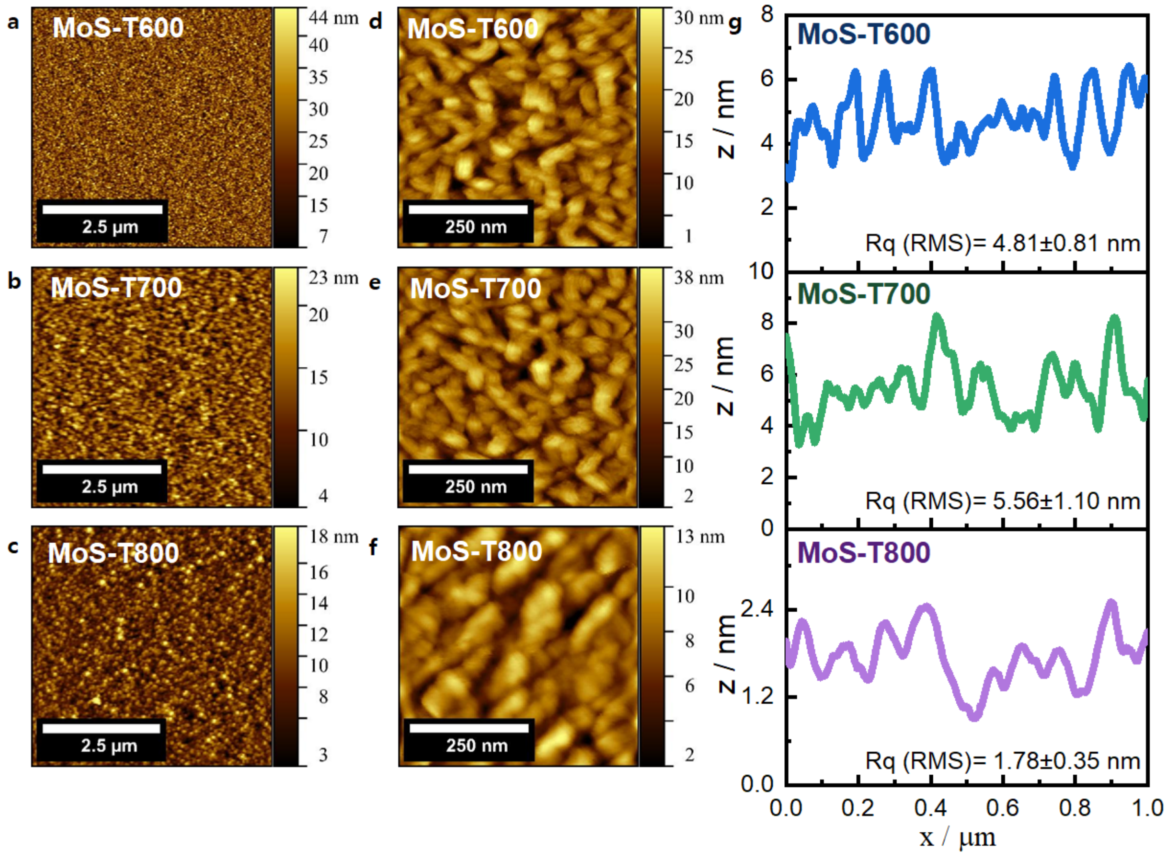

Figure S3. (a-f) AFM topography images showing the large scale (a-c) and small scale (d-f) surface morphology. g Surface roughness comparison of MoS-T600, MoS-T700, and MoS-T800. The height profile and shown surface root mean square roughness *Rq (RMS)* were obtained from the full images (d-f).

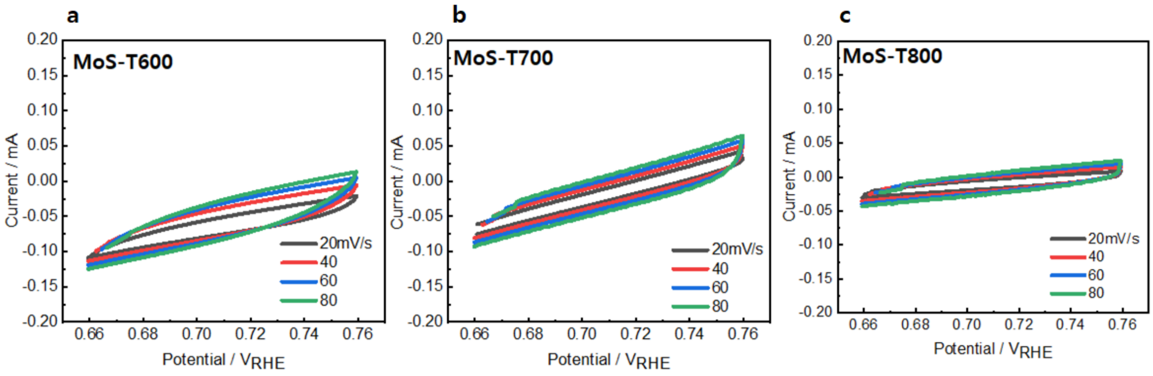

Figure S4. (a-c) Cyclic voltammetry curves of MoS-T600 (a), T700 (b), and T800 (c) measured at various scan rates (20, 40, 60, 80 mV s^{-1}) from 0.66 V to 0.76 V (vs. RHE).

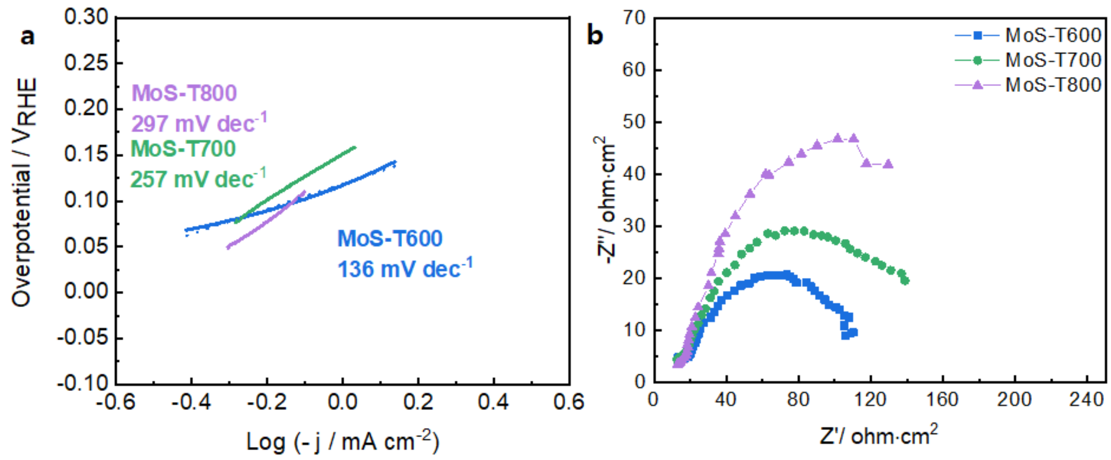

Figure S5. (a, b) Tafel plot and Nyquist plot for MoS_2 electrodes with different annealing temperatures. The EIS measurements were performed at –0.2 V (vs. RHE) using a frequency range of 100 kHz to 0.1 Hz.

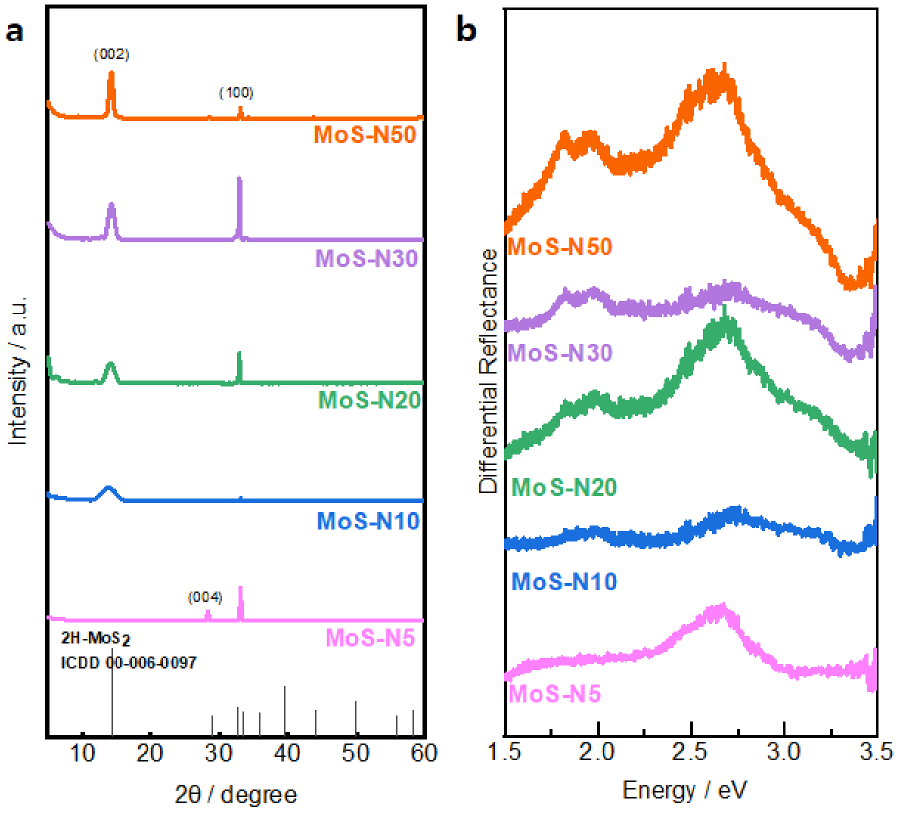

Figure S6. (a, b**)** Structural evolution of MBE-grown MoS_2: XRD patterns and differential reflectance spectra of MoS_2 thin films deposited with 5, 10, 20, 30, and 50 cycles.

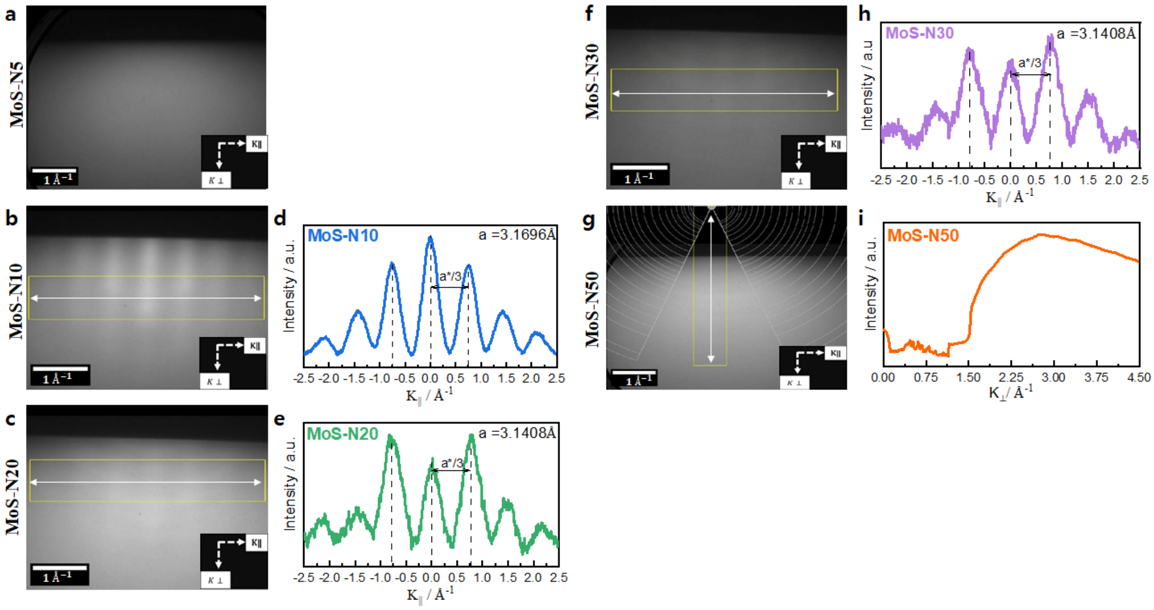

Figure S7. (a, b, c, f, g) RHEED patterns for the MoS-N5(a), N10 (b), N20 (c), N30 (f), and N50 (g). MoS-N5 shows no features, MoS-N10, MoS-N20, MoS-N30 show streaks, and MoS-N50 shows RHEED rings. (d, e, h) Intensity profiles as a function of momentum transfer parallel to the substrate $K_{\parallel}$ along the white line shown in panels (b, c, f). (i) Intensity profiles as a function of momentum transfer perpendicular to the substrate $K_{\perp}$ along the white line shown in panel (g).

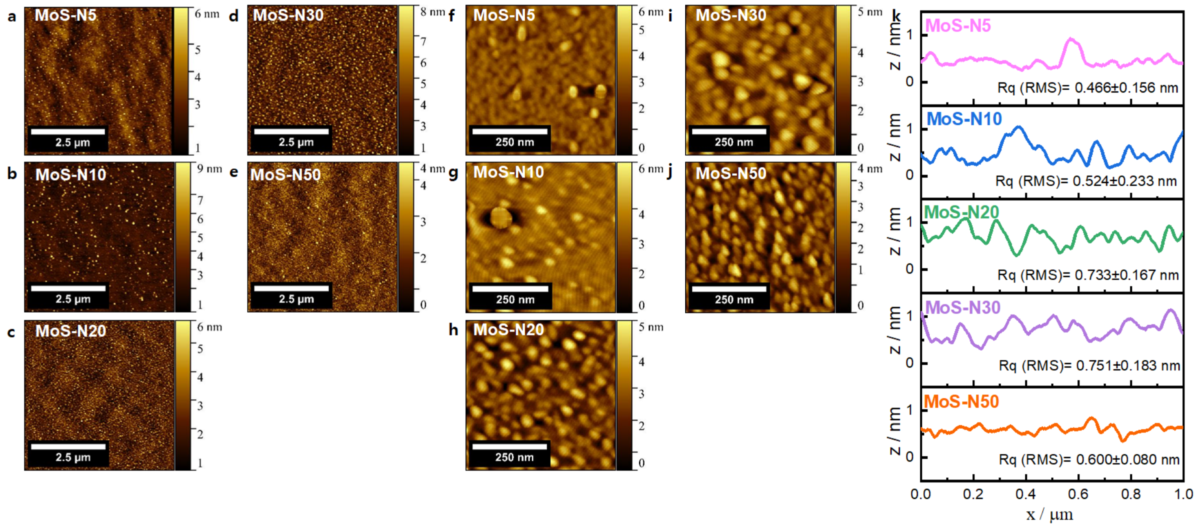

Figure S8. (a-j) AFM topography images showing the large scale (a-e) and small scale (f-j) surface morphology. (k) Surface roughness comparison for MoS-N5, MoS-N10, MoS-N20, MoS-N30, and MoS-N50. The height profile and shown surface root mean square roughness Rq (RMS) were obtained from the full images (f - j).

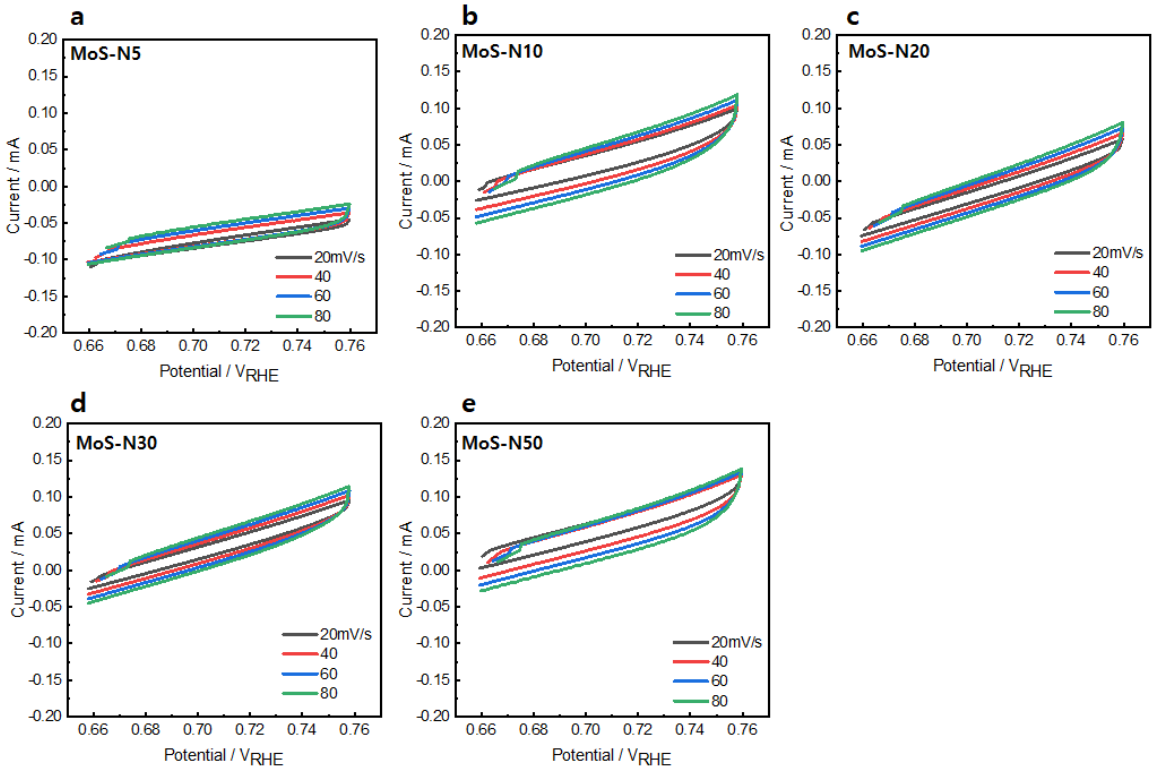

Figure S9. (a-e**)** Cyclic voltammetry curves of MoS-N5 (a), N10 (b), N20 (c), N30 (d), and N50 (e) measured at various scan rates (20, 40, 60, 80 mV s^{-1}) from 0.66 V to 0.76 V (vs. RHE).

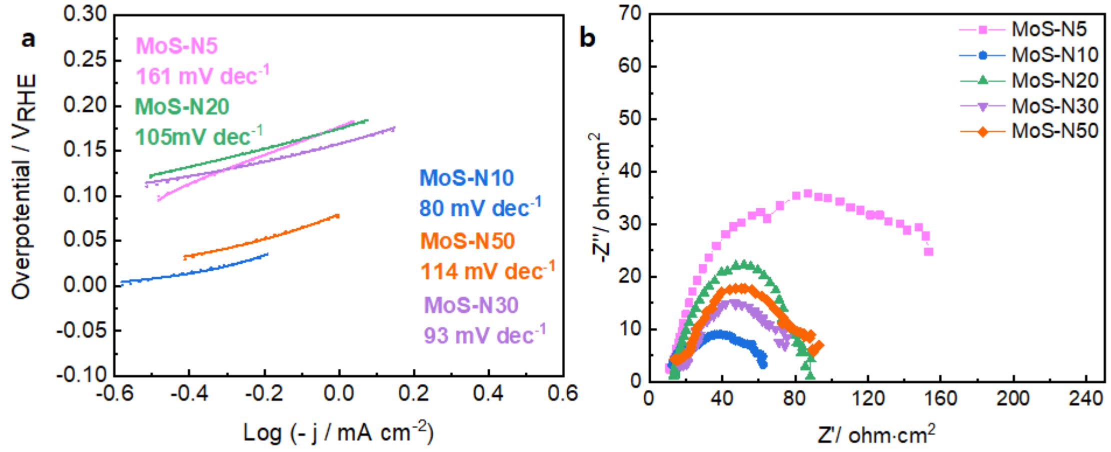

Figure S10. (a, b**)** Tafel plot and Nyquist plot for MoS_2 electrodes with different deposited cycles.

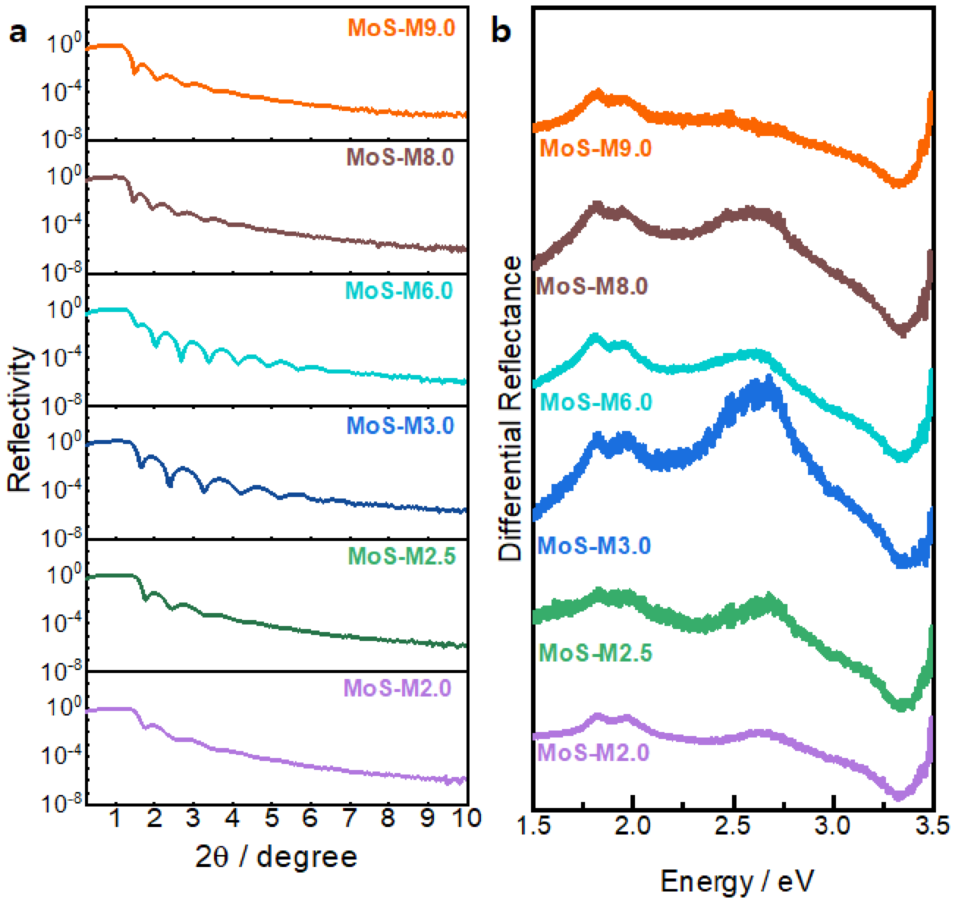

Figure S11. (a, b) Structural evolution of MBE-grown MoS_2: XRR patterns and differential reflectance spectra of MoS_2 thin films grown with varying sulfur thickness (2.0–9.0).

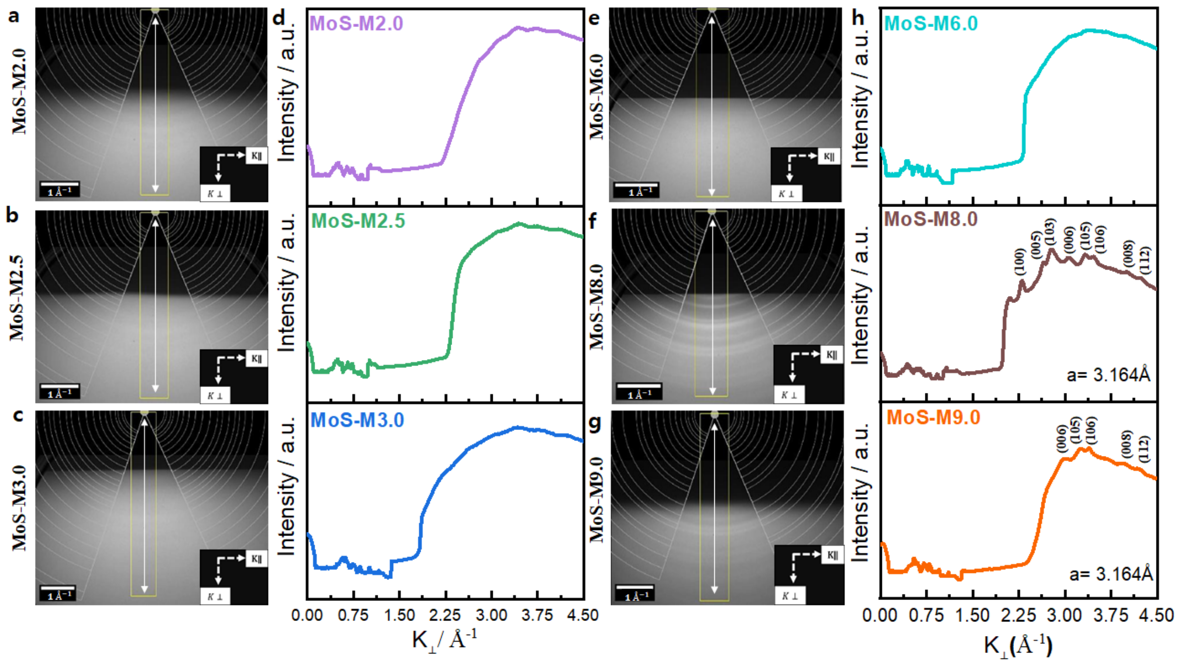

Figure S12. (a, b, c, e, f, g) RHEED patterns for MoS-M2.0 (a), MoS-M2.5 (b), MoS-M3.0 (c), MoS-M6.0 (e), MoS-M8.0 (f), and MoS-M9.0 (g). (d, h) Intensity profiles as a function of momentum transfer perpendicular to the substrate $K_{\perp}$ along the white line shown in panels (a, b, c, e, f and g). The diffraction peaks for MoS-M8.0 (f), and MoS-M9.0 (g) are labeled according to the 2H-MoS_2 structure.

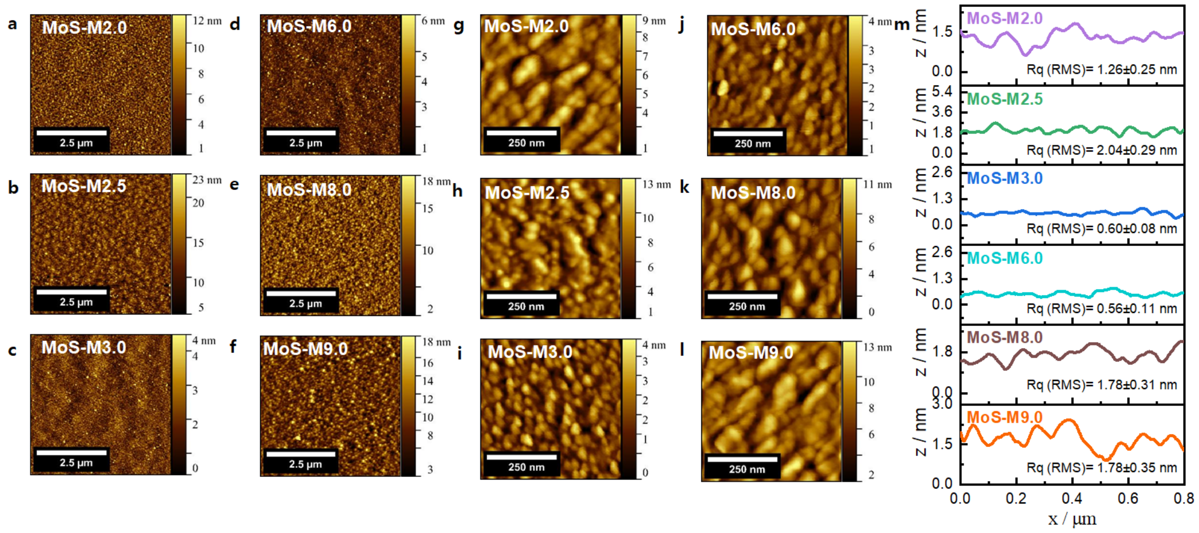

Figure S13. (a-l) AFM topography images showing the large scale and small scale surface morphology for MoS-M2.0 (a, g), MoS-M2.5 (b, h), MoS-M3.0 (c, i), MoS-M6.0 (d, j), MoS-M8.0 (e, k), and MoS-M9.0 (i, l). (m**)** The height profile and shown surface root mean square roughness *Rq (RMS)* were obtained from the full small scale images (g-l).

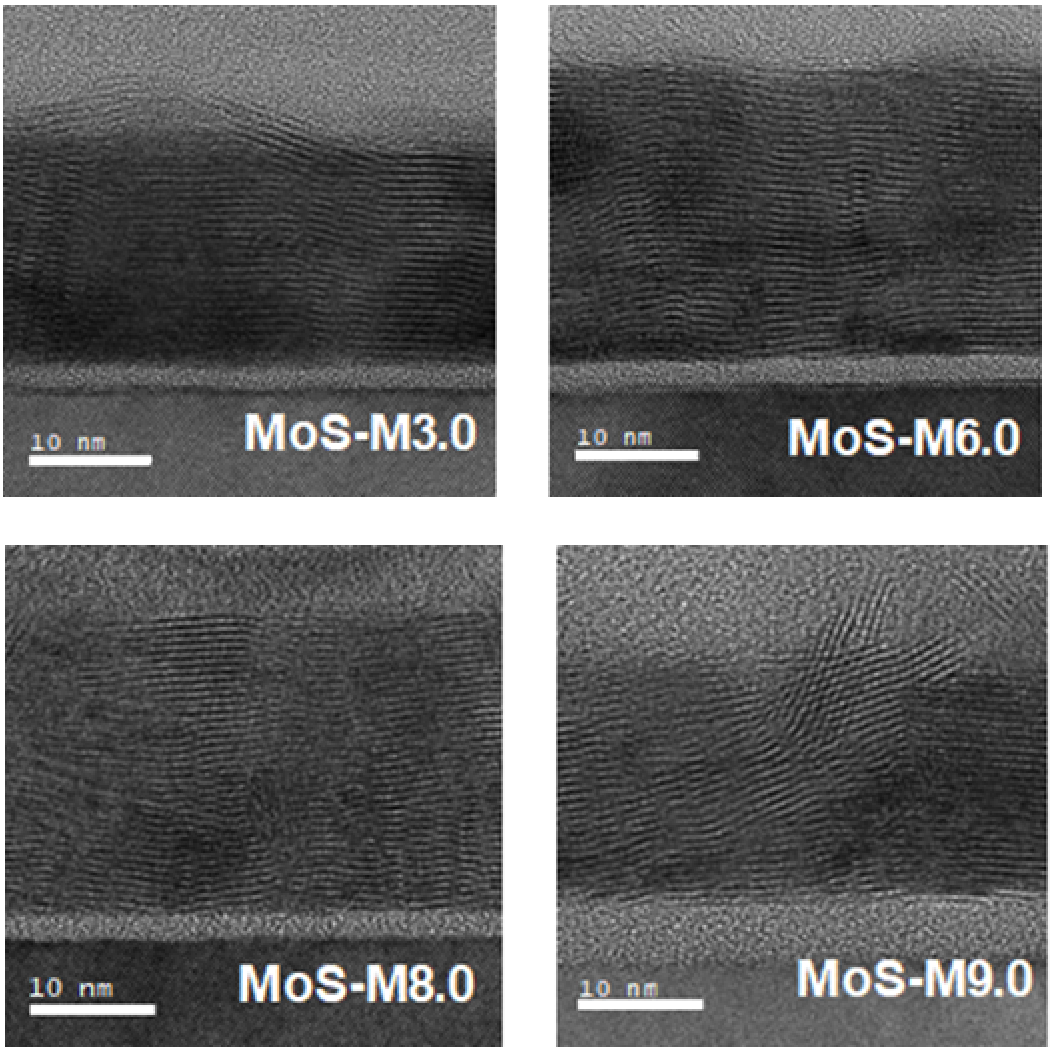

Figure S14. TEM images of MoS_2 films grown with varying sulfur thickness (3.0–9.0).

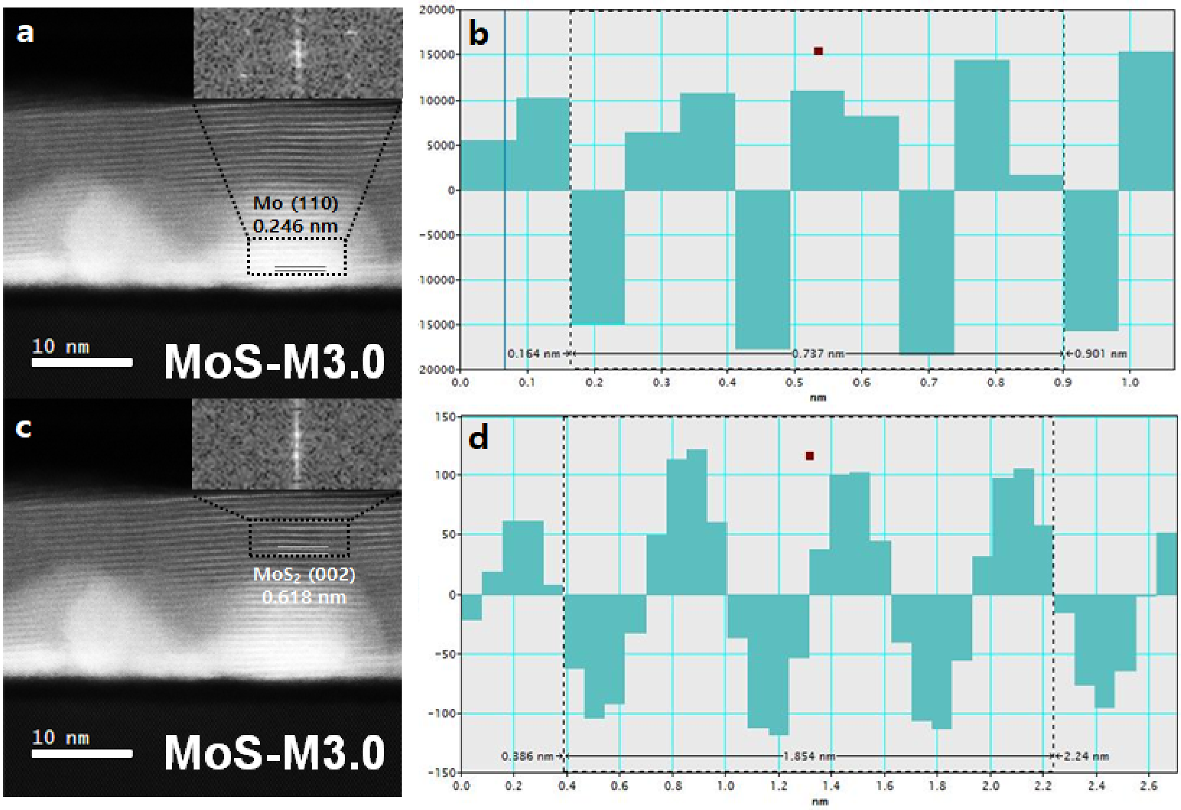

Figure S15. (a) STEM image of MoS–M3.0 with the corresponding fast Fourier transformation (FFT) pattern (inset), showing lattice fringes of the Mo (110) plane (0.246 nm). (b) Inverse fast Fourier transformation (IFFT) profile from (a), confirming the spacing. (c) STEM image of MoS–M3.0 with lattice fringes of the MoS_2 (002) plane (0.618 nm); inset shows the FFT pattern. (d) IFFT profile from (c), showing periodic fringes consistent with MoS_2 (002).

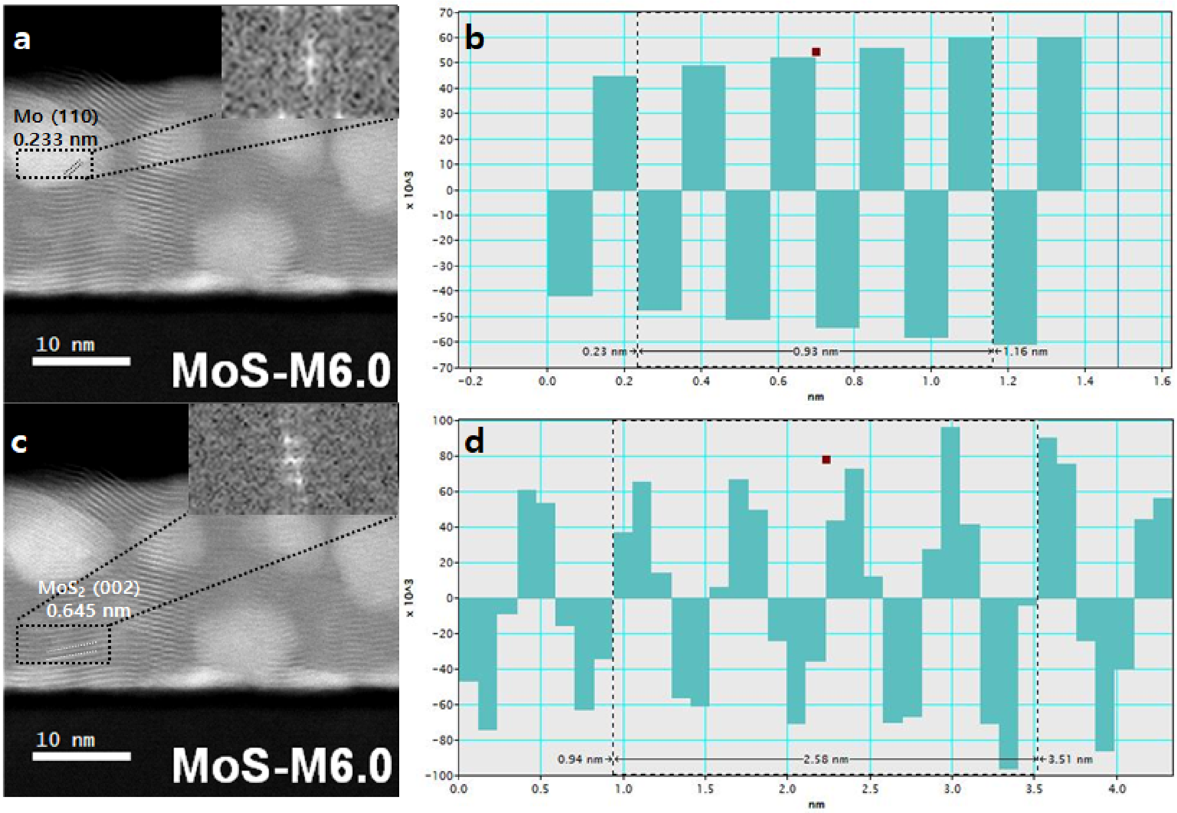

Figure S16. (a) STEM image of MoS–M6.0 with the corresponding FFT pattern (inset), showing lattice fringes of the Mo (110) plane (0.233 nm). **(**b) IFFT profile from (a), confirming the spacing. (c) STEM image of MoS–M6.0 with lattice fringes of the MoS_2 (002) plane (0.645 nm); inset shows the FFT pattern. (d) IFFT profile from (c), showing periodic fringes consistent with MoS_2 (002).

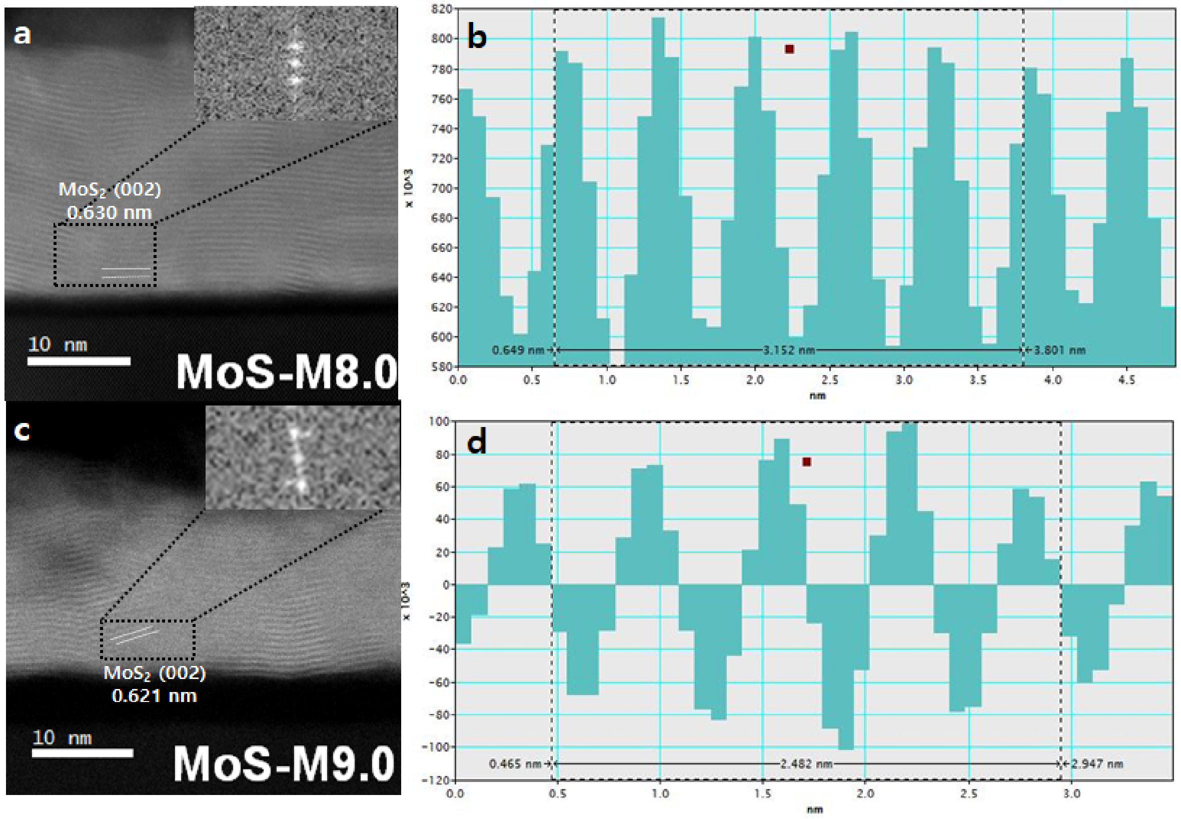

Figure S17. (a) STEM image of MoS–M8.0 with the corresponding FFT pattern (inset), showing lattice fringes of the MoS_2 (002) plane (0.630 nm). (b) IFFT profile from (a), confirming the spacing. (c) STEM image of MoS–M9.0 with lattice fringes of the MoS_2 (002) plane (0.621 nm); inset shows the FFT pattern. (d) IFFT profile from (c), showing periodic fringes consistent with MoS_2 (002).

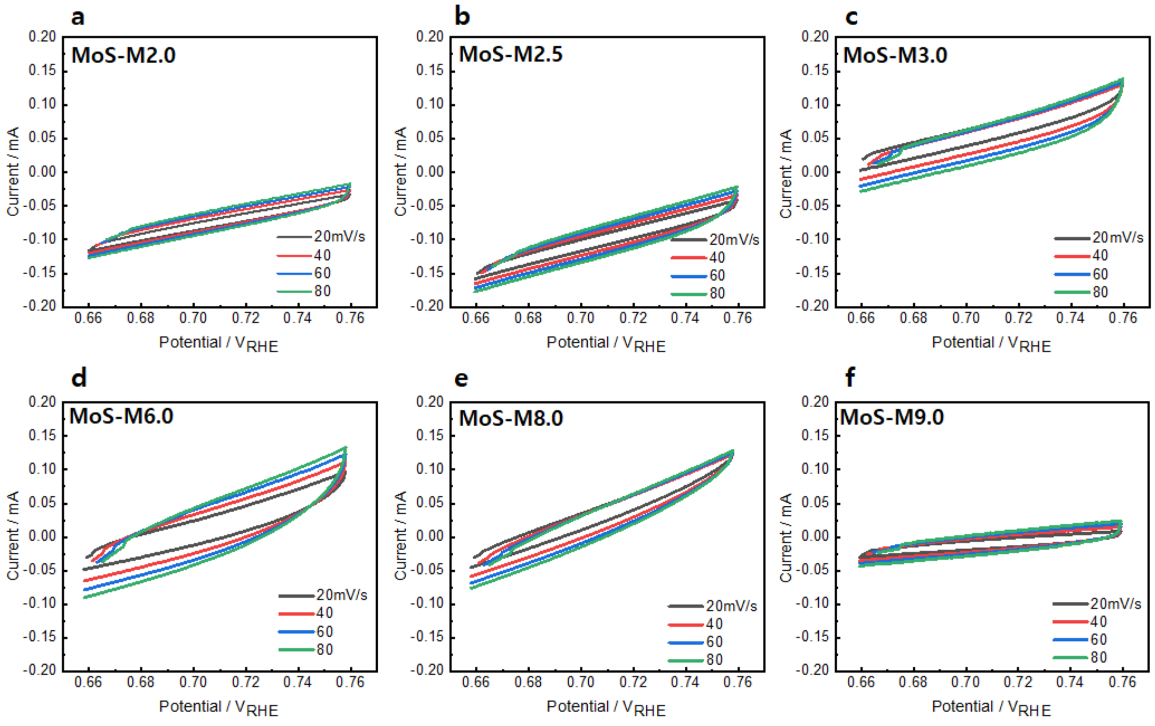

Figure S18. (a-f) Cyclic voltammetry curves of MoS-M2.0 (a), M2.5 (b), M3.0 (c), M6.0 (d), M8.0 (e), and M9.0 (f) measured at various scan rates (20, 40, 60, 80 mV s^{-1}) from 0.66 V to 0.76 V (vs. RHE).

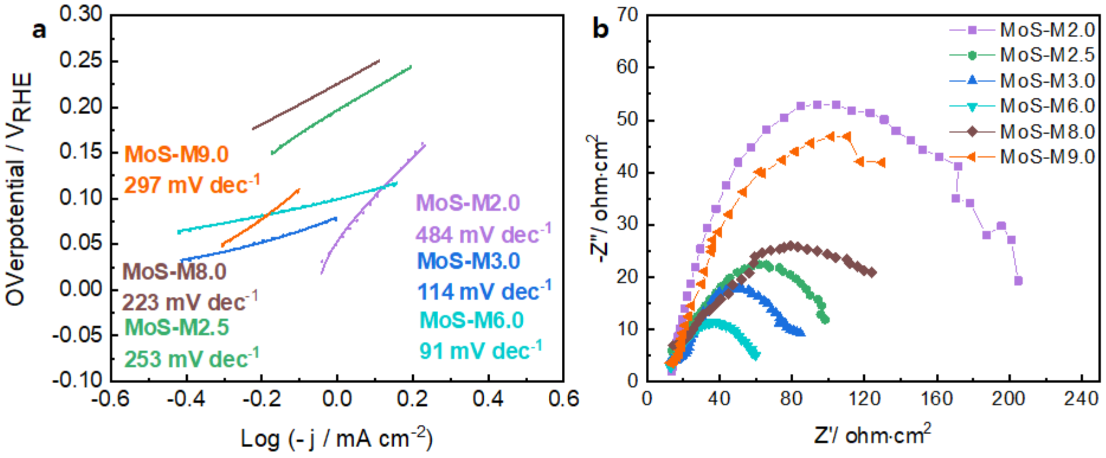

Figure S19. (a, b) Tafel plot and Nyquist plot for MoS_2 electrodes with different S thickness.

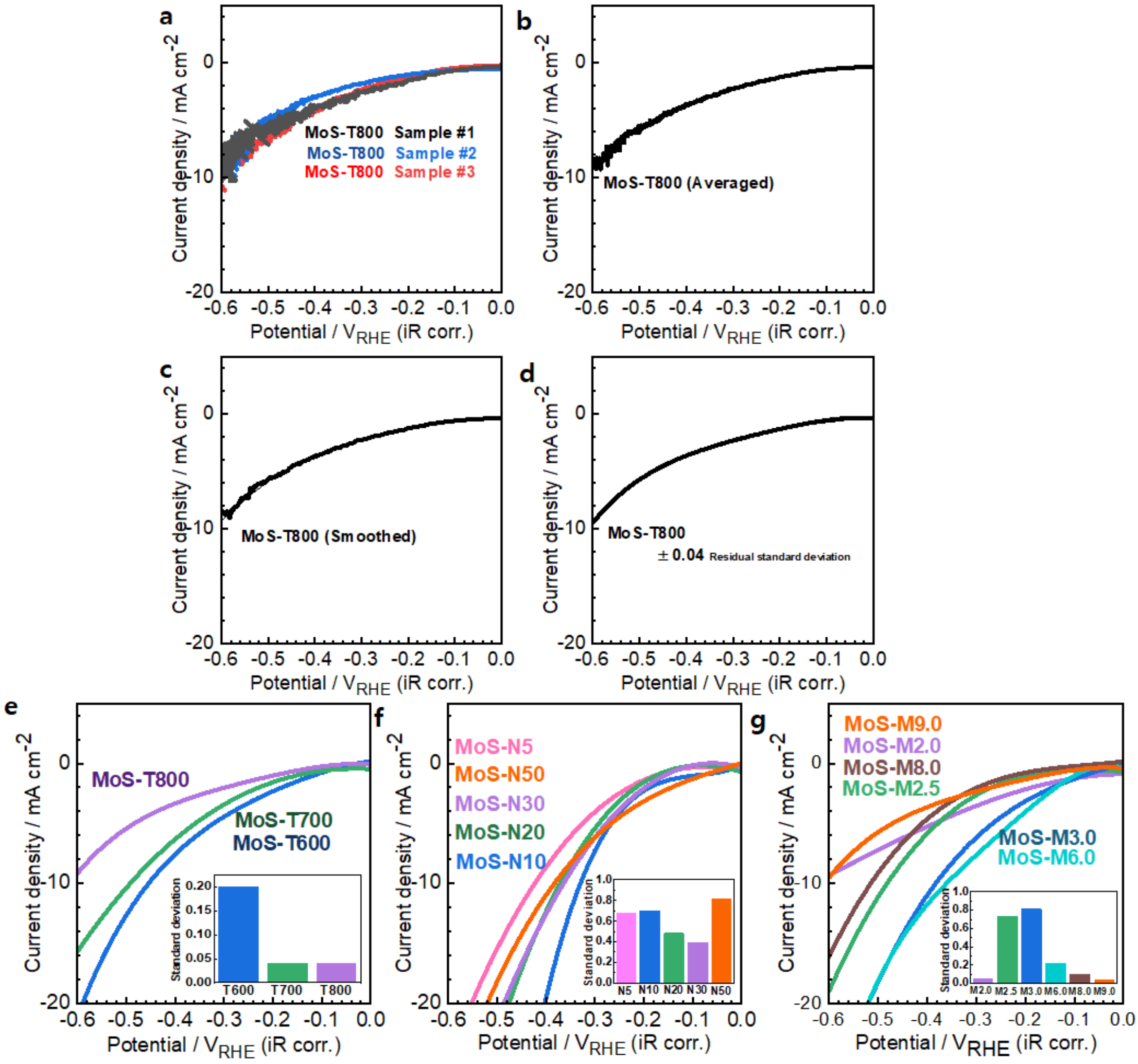

Figure S20. Data processing workflow for HER LSV polarization curves of MoS–T800: (a) Three independently measured HER LSV curves. (b) Averaged polarization curve obtained from the three measurements. (c) Smoothed LSV curve derived from the average data. (d) Trend curve with the residual standard deviation indicating the noise level of the averaged LSV. (e-g) Trend curves of MoS_2 films with the residual standard deviation.

Table S1. Comparison of HER performance metrics of MBE-grown MoS_2 with representative MoS_2-based catalysts reported in the literature

Catalyst	Synthesis method	Substrate	Electrolyte	Loading amount / μg cm^{-2}	Mass-based current density at -0.25V_{RHE} / mA mg^{-1}	MoS_2 mass-based TOF at -0.25V_{RHE} / nmol H_2 $\mu g^{-1}s^{-1}$	η at -10 mA cm^{-2} / V_{RHE}	Rct / Ohm·cm^2	Tafel slope / mV dec^{-1}	Ref.
**MoS-N10**	**Molecular beam epitaxy**	**Si wafer**	**1M KOH**	**3.7**	**1019**	**5.28**	**-0.33**	**52.8**	**80**	**This work**
FeP/MoS_2	Hydrothermal synthesis	Glassy carbon	0.5M H_2SO_4	1000	90	0.46	-0.11	33	67.8	[S1]
MoS_2/CC	Hydrothermal synthesis	Carbon cloth	0.5M H_2SO_4	190	452	2.34	-	-	50	[S2]
rGO/MoS_2-S	Hydrothermal synthesis	Glassy carbon	1M H_2SO_4	2000	5	0.03	-0.25	80	75 ±4	[S3]
MoS_2/FNS/FeNi foam	Chemical Vapour Deposition	FeNi foam	1M KOH	150	565	2.93	-0.12	4.0	45.1	[S4]
MoS_2/IPC-2	Hydrothermal synthesis	Glassy carbon	0.5M H_2SO_4	254	886	4.59	-0.18	10.1	38	[S5]
Exfoliated 3R MoS_2	Exfoliation	Glassy carbon	0.5M H_2SO_4	106	94	0.49	-0.25	-	58	[S6]
MoS_2–TMS_2–$TMoO_x$ hybrids	Hydrothermal synthesis	Glassy carbon	1M KOH	200	50	0.26	-0.25	-	74	[S7]
MoS_2/ GO 50/1 550◦C	Impinging jet reactor.	Glassy carbon	0.5M H_2SO_4	1131	19.6	0.10	-0.22	-	84.3	[S8]
Rh-MoS_2	Lithium intercalation synthesis	Glassy carbon	0.5M H_2SO_4	309	13870	7186.04	-0.05	5.1	24	[S9]
MoS_2/SWNT	Exfoliation	PyC	0.5M H_2SO_4	1450	5.31	0.03	-	72	102±17	[S10]

References

S1. Suliman, M. H.; Adam, A.; Li, L.; Tian, Z.; Siddiqui, M. N.; Yamani, Z. H.; Qamar, M. FeP/MoS_2 Enriched with Dense Catalytic Sites and High Electrical Conductivity for the Hydrogen Evolution Reaction. ACS Sustain. Chem. Eng. **2019**, 7 (21), 17671–17681. https://doi.org/10.1021/acssuschemeng.9b03799.

S2. Yan, Y.; Xia, B.; Li, N.; Xu, Z.; Fisher, A.; Wang, X. Vertically Oriented MoS_2 and WS_2 Nanosheets Directly Grown on Carbon Cloth as Efficient and Stable Three-Dimensional Hydrogen-Evolving Cathodes. J. Mater. Chem. A **2015**, 3, 131–135. https://doi.org/10.1039/C4TA04858J.

S3. Kamila, S.; Mohanty, B.; Samantara, A. K.; Guha, P.; Ghosh, A.; Jena, B.; Satyam, P. V.; Mishra, B. K.; Jena, B. K. Highly Active 2D Layered MoS_2–rGO Hybrids for Energy Conversion and Storage Applications. Sci. Rep. **2017**, 7, 8378. https://doi.org/10.1038/s41598-017-08677-5.

S4. Wu, Y.; Li, F.; Chen, W.; Xiang, Q.; Ma, Y.; Zhu, H.; Tao, P.; Song, C.; Shang, W.; Deng, T.; Wu, J. Coupling Interface Constructions of MoS_2/$Fe_5Ni_4S_8$ Heterostructures for Efficient Electrochemical Water Splitting. Adv. Mater. **2018**, 30, 1803151. https://doi.org/10.1002/adma.201803151.

S5. Ko, D.; Jin, X.; Seong, K.-D.; Yan, B.; Chai, H.; Kim, J. M.; Hwang, M.; Choi, J.; Zhang, W.; Piao, Y. Few-Layered MoS_2 Vertically Aligned on Three-Dimensional Interconnected Porous Carbon Nanosheets for Hydrogen Evolution. Appl. Catal. B **2019,** 248, 357–365. https://doi.org/10.1016/j.apcatb.2019.02.035.

S6. Luxa, J.; Spejchalová, L.; Jakubec, I.; Sofer, Z. MoS_2 Stacking Matters: 3R Polytype Significantly Outperforms 2H MoS_2 for the Hydrogen Evolution Reaction. Nanoscale **2021**, 13, 19391–19398. https://doi.org/10.1039/D1NR03284D.

S7. Wang, J.; Li, L.; Wang, L.; Liu, Y.; Sun, W.; Li, W.; Li, G. In Situ Growth of MoS_2 Nanosheet Arrays and TS_2 (T = Fe, Co, and Ni) Nanocubes onto Molybdate for Efficient Oxygen Evolution Reaction and Improved Hydrogen Evolution Reaction. ACS Omega **2018**, 3, 464–471. https://doi.org/10.1021/acsomega.7b01965.

S8. Bojarska, Z.; Mazurkiewicz-Pawlicka, M.; Mierzwa, B.; Plocinski, T.; Makowski, L. Effect of the Carbon Support on MoS_2 Hybrid Nanostructures Prepared by an Impinging Jet Reactor for Hydrogen Evolution Reaction Catalysis. J. Environ. Chem. Eng. **2022**, 10, 108038. https://doi.org/10.1016/j.jece.2022.108038.

S9. Cheng, Y.; Lu, S.; Liao, F.; Liu, L.; Li, Y.; Shao, M. Rh–MoS_2 Nanocomposite Catalysts with Pt-Like Activity for Hydrogen Evolution Reaction. Adv. Funct. Mater. **2017**, 27 (23), 1700359. https://doi.org/10.1002/adfm.201700359.

S10. McAteer, D.; Gholamvand, Z.; McEvoy, N.; Harvey, A.; O'Malley, E.; Duesberg, G. S.; Coleman, J. N. Thickness Dependence and Percolation Scaling of Hydrogen Production Rate in MoS_2 Nanosheet and Nanosheet–Carbon Nanotube Composite Catalytic Electrodes. ACS Nano **2016**, 10 (1), 672–683. https://doi.org/10.1021/acsnano.5b05907.
